# Siamese model for collateral score prediction from computed tomography angiography images in acute ischemic stroke

**DOI:** 10.3389/fnimg.2023.1239703

**Published:** 2024-01-11

**Authors:** Valerio Fortunati, Jiahang Su, Lennard Wolff, Pieter-Jan van Doormaal, Jeanette Hofmeijer, Jasper Martens, Reinoud P. H. Bokkers, Wim H. van Zwam, Aad van der Lugt, Theo van Walsum

**Affiliations:** ^1^Quantib BV, Rotterdam, Netherlands; ^2^Biomedical Imaging Group Rotterdam, Department of Radiology & Nuclear Medicine, Erasmus MC, University Medical Center Rotterdam, Rotterdam, Netherlands; ^3^Department of Radiology & Nuclear Medicine, Erasmus MC, University Medical Center Rotterdam, Rotterdam, Netherlands; ^4^Clinical Neurophysiology, MIRA Institute for Biomedical Technology and Technical Medicine, University of Twente, Enschede, Netherlands; ^5^Department of Neurology, Rijnstate Hospital, Arnhem, Netherlands; ^6^Department of Radiology and Nuclear Medicine, Rijnstate Hospital, Arnhem, Netherlands; ^7^Faculty of Medicine, University Medical Center Groningen, Groningen, Netherlands; ^8^Department of Radiology & Nuclear Medicine, Maastricht UMC, Cardiovascular Research Institute Maastricht, Maastricht, Netherlands

**Keywords:** acute ischemic stroke, CTA, collateral score, end-to-end classification, Siamese model

## Abstract

**Introduction:**

Imaging biomarkers, such as the collateral score as determined from Computed Tomography Angiography (CTA) images, play a role in treatment decision making for acute stroke patients. In this manuscript, we present an end-to-end learning approach for automatic determination of a collateral score from a CTA image. Our aim was to investigate whether such end-to-end learning approaches can be used for this classification task, and whether the resulting classification can be used in existing outcome prediction models.

**Methods:**

The method consists of a preprocessing step, where the CTA image is aligned to an atlas and divided in the two hemispheres: the affected side and the healthy side. Subsequently, a VoxResNet based convolutional neural network is used to extract features at various resolutions from the input images. This is done by using a Siamese model, such that the classification is driven by the comparison between the affected and healthy using a unique set of features for both hemispheres. After masking the resulting features for both sides with the vascular region and global average pooling (per hemisphere) and concatenation of the resulting features, a fully connected layer is used to determine the categorized collateral score.

**Experiments:**

Several experiments have been performed to optimize the model hyperparameters and training procedure, and to validate the final model performance. The hyperparameter optimization and subsequent model training was done using CTA images from the MR CLEAN Registry, a Dutch multi-center multi-vendor registry of acute stroke patients that underwent endovascular treatment. A separate set of images, from the MR CLEAN Trial, served as an external validation set, where collateral scoring was assessed and compared with both human observers and a recent more traditional model. In addition, the automated collateral scores have been used in an existing functional outcome prediction model that uses both imaging and non-imaging clinical parameters.

**Conclusion:**

The results show that end-to-end learning of collateral scoring in CTA images is feasible, and does perform similar to more traditional methods, and the performance also is within the inter-observer variation. Furthermore, the results demonstrate that the end-to-end classification results also can be used in an existing functional outcome prediction model.

## 1 Introduction

According to the World Health Organization (WHO), stroke is the second leading cause of death and an important cause of disability.^1^ As such, it is a major challenge for the health care system. The large majority of strokes is ischemic, caused by an occlusion of one of the cerebral arteries. Traditionally, such strokes have been treated by the intra-venous administration of tPA to dissolve the blood cloth causing the occlusion. More recently, several large trials demonstrated that intra-arterial approaches to remove the clot, either by dissolving it with thrombolytic agents, or by explicitly removing the clot (mechanical thrombectomy) are effective procedures for ischemic stroke patients (Goyal et al., 2016). However, not all patients equally benefit from intra-arterial treatment, a procedure which is not without risks. Consequently, much research has been devoted to determining which factors may best predict successful treatment outcome.

The collateral circulation, i.e., alternative routes for blood supply to the brain region affected by the stroke, is assumed to play an important role in ischemic stroke (Liebeskind, 2003). It is known that collateral status is a modifier of the effect of endovascular treatment on functional outcome (Berkhemer et al., 2016). Collateral circulation in a stroke patient is generally visualized using Computed Tomography Angiography (CTA) imaging. CTA is a medical imaging technique that uses X-ray technology and computed tomography technique to visualize blood vessels throughout the body. For stroke either single or multiphase CTA is used: in single phase CTA one image is acquired, whereas in multiphase CTA multiple images are acquired, with fixed delays, so as to image, e.g., the arterial and venous phase. Assessing collateral circulation and investigating the effect of collateral status on treatment outcome requires a scoring scheme for collaterals. Several (manual) grading scores have been developed in the past for collateral scoring (McVerry et al., 2012), among which a four point scale (Tan et al., 2009), five point scales (Maas et al., 2009; Souza et al., 2012), and a ten point scale (Menon et al., 2013) for single phase CTA, and a six point scale (Menon et al., 2015a) for multiphase CTA (Menon et al., 2015b). All these approaches are based on a visual assessment of the vessels present in the affected hemisphere compared to the same regions in the contralateral hemisphere.

Stroke patients benefit from fast treatment, as “time is brain.” Human collateral scoring in clinical practice may be time consuming, and thus potentially delay treatment. In addition, there is a large inter observer variation when scored by humans (Tan et al., 2009; McVerry et al., 2012; Su et al., 2020). Automation of the scoring thus may be relevant, firstly by not requiring human intervention, and secondly, it may be a more objective (and quantitative) measure, and thus provide better results in outcome prediction. Consequently, automated collateral scoring has been developed and reported. Boers et al. (2017) reported a collateral scoring method for single phase CTA that uses a Hessian-based vesselness filter for detecting vessels, and subsequently quantifies the amount of vessels in both hemispheres using an atlas-based mask of the territory at risk. This image mask was created by registration and combination of stroke infarct lesions from many patients to a healthy individual, and thresholding the probability map obtained at 5%. Additionally, they assessed the value of this quantitative collateral scoring by comparing it to manual collateral scores for a large set of patients, and they verified that the score could be used in functional outcome prediction (Boers et al., 2018). Su et al. (2020) described a collateral scoring approach where vessels were segmented using a U-Net. Features were subsequently derived by comparing lengths, volume and intensity of the segmented vessels inside the middle cerebral artery (MCA) territory (determined from an atlas with vessel information; Peter et al., 2017), and these features were used as input for a random forest classifier to compute a quantitative collateral score. Several commercial solution have appeared as well, generally lacking detailed information on the underlying algorithms. Grunwald et al. (2019) assessed the e-STROKE SUITE of Brainomix Ltx, which follows a similar strategy of vessel segmentation and quantification in the MCA territory.

Collateral scoring depends on accurate timing of the imaging, as imaging in early arterial or late venous phase may lead to incorrect scores. Therefore, collateral scoring on multi phase CTA or 4D CTA has also been investigated. Aktar et al. (2020) follow an approach that is similar to the approaches of Boers et al. (2018) and Su et al. (2020): comparing presence of vessels in the affected brain vs. the full vasculature. The vessel information was extracted from the 4D CTA using fast robust matrix completion on a cohort of healthy subjects and the patient, where the patient's unfilled vessels and the estimated full vasculature were modeled as sparse and low-rank components, respectively.

To the best of our knowledge, all automated collateral scoring on CTA images follow a procedure that is similar to how humans perform this task: comparing the amount of arteries in the affected and contralateral hemisphere (or using a healthy reference subject). We are not aware of any approach to directly predict collateral score from CTA image using AI approaches. However, currently, AI approaches, and more specifically CNN-based approaches, are dominating the field of medical imaging. Especially the segmentation task seems to be well suited to the CNN paradigm. The U-net is a well-known example, and in the original U-net paper Ronneberger et al. (2015) demonstrated that augmentation helps to get good results even in case of little training data. Isensee et al. (2020) later showed that a basic U-net is able to address a large variety of medical imaging segmentation tasks, and that even the configuration can be automatically determined. Segmentation clearly is a task that well matches the CNN approach (Isensee et al., 2020). There are several reasons for this. First, the appearance of the image is often directly linked to the segmentation result. In addition, the ability to augment both the image and the segmentation allows to generate a much larger training set easily.

More advanced approaches have been described to perform more complex tasks, such as the prediction of brain lesions in follow-up MR images based on baseline MR images, as is reported by the Ischemic Stroke Lesion Segmentation (ISLES) challenges organizers (Winzeck et al., 2018). Whereas, the trend is toward deep learning approaches for such tasks, the results for such tasks are generally less accurate than segmentation tasks. In the brain lesion prediction, e.g., mean Dice scores of 0.2–0.3 were obtained. The reasons for such relatively low scores (e.g., compared to a standard CNN-based segmentation) may be in the complexity of the task, and the limited availability of training data.

Similarly, direct quantification of biomarkers, or prediction of outcome is a relevant task in the field of stroke, which may be addressed with deep learning based methods. A recent example is classifying CTA images into stroke and non-stroke cases (Barman et al., 2019). For this application, a Siamese network approach was followed, and an area under the curve of ~0.9 was obtained for various scenarios. Whereas, apparently detection of stroke is feasible, we are interested in investigating to what extent networks can be trained to directly predict more advanced biomarkers or even treatment outcome. Similar to the superiority of neural network based segmentation approaches over traditional ones, such classification and prediction tasks may also outperform traditional methods.

The purpose of our study is therefore to investigate to what extent collateral scoring can be directly performed using neural networks, and whether this can be trained in an end-to-end manner. In addition, we want to assess whether such collateral scores have any added value in existing functional outcome prediction models. The motivation for this work is twofold. First, it is clinically relevant to provide collateral scores that can be used in therapeutic decision making. Second, the neural network architecture developed and assessed for this purpose, may be the basis for future studies that investigate prediction of functional outcome directly on the images.

Our contributions are three-fold:

1. we developed a dedicated end-to-end architecture for collateral scoring, that is adapted to the task of processing stroke patient images;
2. we assessed the method on a large set of stroke patients, comparing it to human observers;
3. we assessed the collateral scoring approach in an existing functional outcome prediction model.

## 2 Methods and materials

### 2.1 Method

#### 2.1.1 Method motivation

**Figure 1:**
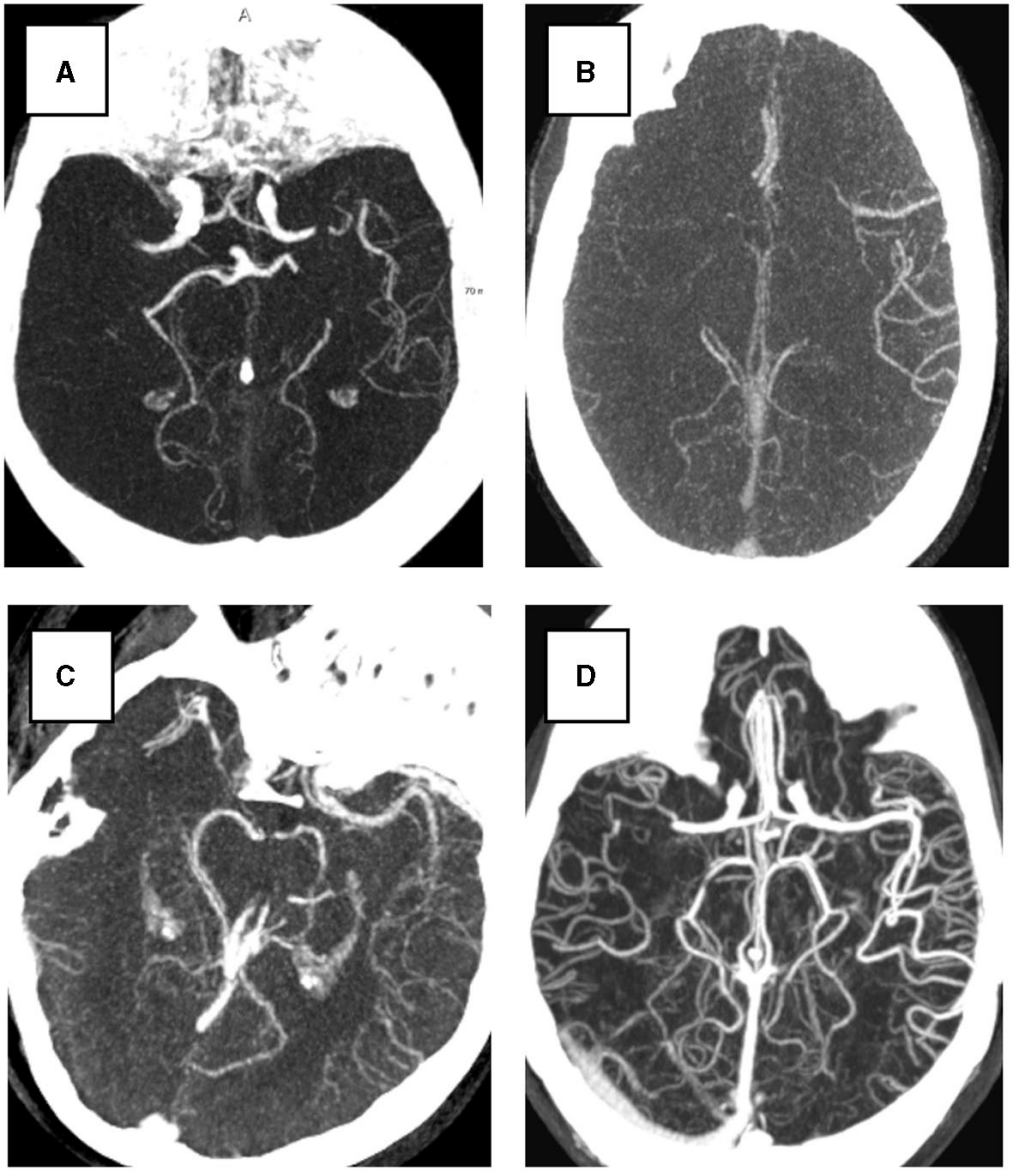
Examples of four collateral score in Maximum Intensity Projection (MIP) from 3D CTA image. All subjects have a right hemisphere occlusion. **(A)** Collateral score 0; **(B)** Collateral score 1; **(C)** Collateral score 2; **(D)** collateral score 3.

The collateral score was designed to be performed by a human observer. The semi-quantitative nature of the score helps clinicians to translate the image features to a number. The score is deduced by comparing the MCA regions, i.e., the brain region fed by the left and right MCA, in both hemispheres of the patient's brain. The vessels in the occluded territory of the affected hemisphere are observed and compared to the contra-lateral hemisphere to visually quantify the collaterals and deduce the four-valued score (Tan et al., 2009; see Figure 1): when the occluded territory is not filled by any collateral vessel a score of 0 is assigned to the patient; when less than 50% of the territory is filled with blood vessels the score is 1; when the territory is filled between 50 and 100% with vessels the assigned score is 2; and when the occluded territory is completely vascularized then the collateral score for the patient is 3. In practice, it has been observed that the dichotomized version of the score (grouping scores 0 and 1 to a score of 0, and 2 and 3 to a score of 1) is clinically the most relevant. In our method we mimic the human visual assessment procedure using a Siamese Neural Network (SNN). SNNs were introduced in the early 90's (Bromley et al., 1993) for signature recognition. In this early seminal work the goal was to discriminate between authentic and forged signatures acquired using a pen input tablet. Generally a Siamese network consists of two identical sub-networks joined at their outputs. During training the two sub-networks extract features from the inputs and the subsequent joining layers measure the difference between the features extracted by the sub-networks. These difference can be used to generate the desired output: in case of signatures, a binary output reporting the signature authenticity. With the advent of deep learning, this idea has been applied to deep neural networks for a multitude of applications (Chicco, 2021). When designing our method we were mostly inspired by the work of Antony et al. (2017) and Tiulpin et al. (2018) who used SNNs to automatically assess Kellgren-Lawrence (KL) scores from X-ray images of the knee. Similar to the collateral score, the KL score is a semi-quantitative score. It is used for a different clinical application that is the quantification of the severity of osteoarthritis of the knee. Similar to their work our method applies the SNN methodology to integrate the symmetric nature of the problem in the architecture of the neural network.

#### 2.1.2 Method overview

**Figure 2:**
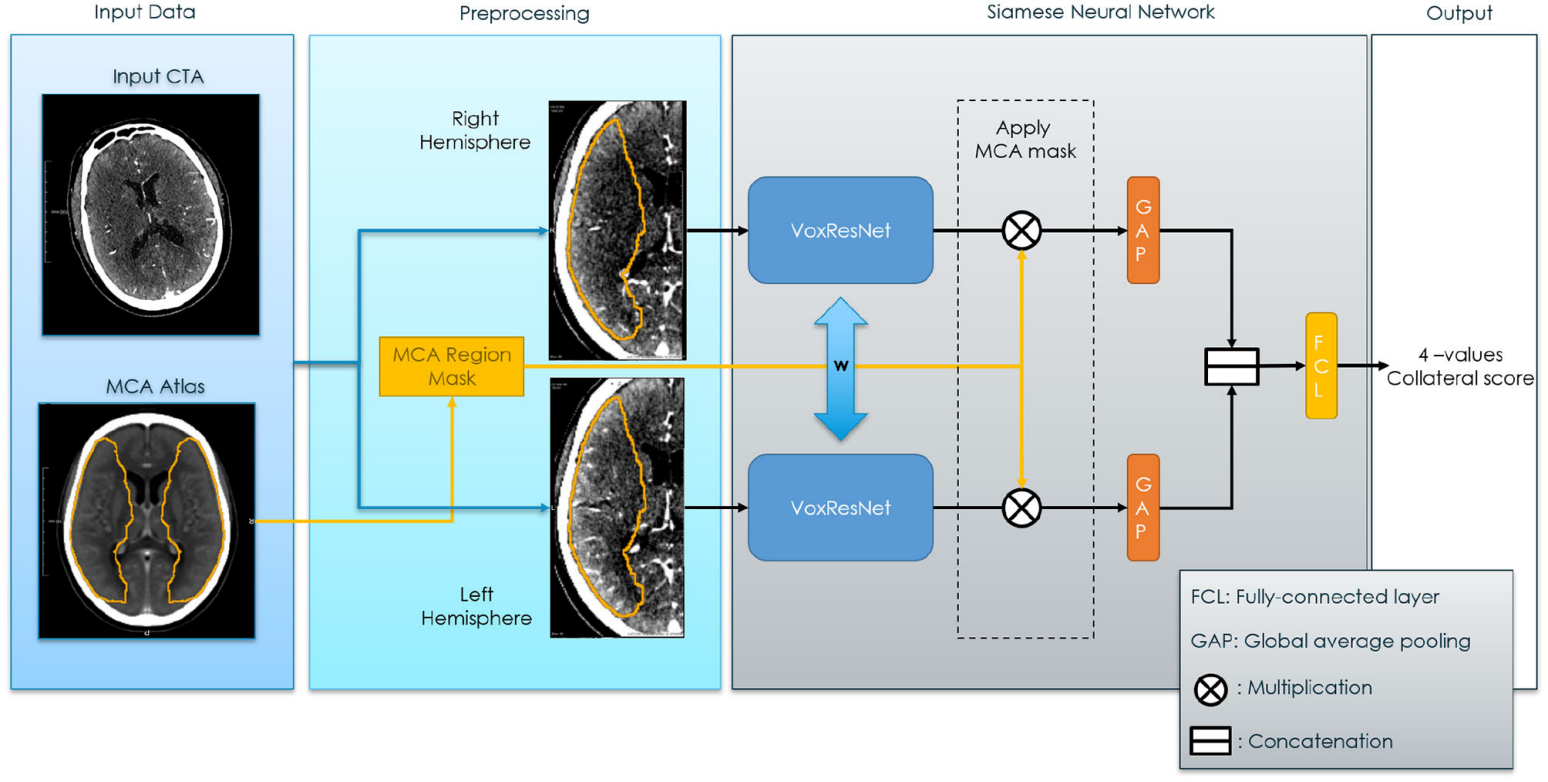
Siamese Neural Network for collateral score prediction. The blue bi-directional arrows between the Voxel Residual Networks indicates weights sharing applied to all the neurons in the neural networks.

In the proposed method, the affected and the healthy hemispheres are used as inputs for the Siamese sub-networks as shown in Figure 2. Then, the feature activation of these sub-networks is joined to obtain the Collateral Score. Since the Collateral Score applies at the subject image level, the entire image needs to be fed to the SNN model for each prediction. In our case the images consists of a high-resolution three-dimensional CTA and therefore, to fit them into a fixed region in the voxel space we needed to pre-processed them. The neural network was based on the VoxResNet (Chen et al., 2018) model architecture which provides a memory efficient multi scale representation of the image. The output activations of the network are then used to obtain the final classification. In a post-processing step the operating point of the classifier was adjusted to match the expected human performance obtained from the inter-observer. The details on the pre-processing, network architecture^2^ and post-processing are given in the following sections.

#### 2.1.3 Image pre-processing

**Figure 3:**
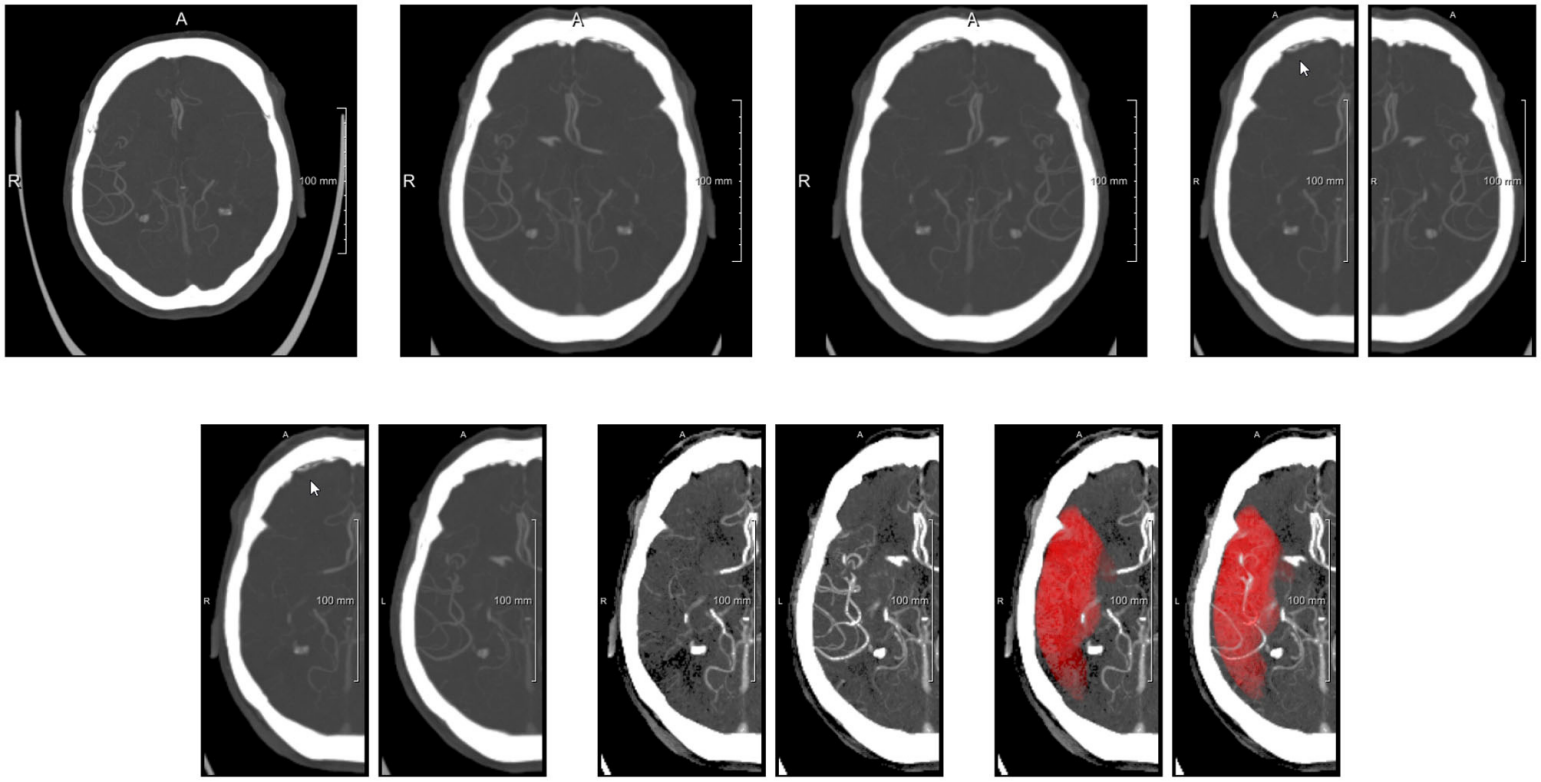
Preprocessing steps visualized with axial MIPs. **(Top row)** From left to right: original image, image after alignment to atlas space (affected side is left side of patient, i.e., right side of image), image after flipping to get affected side at patient right, images of both hemispheres after splitting. **(Bottom row)** Images after flipping non-affected healthy side, images after intensity clipping, and images with MCA mask overlayed (MIP of binarized mask).

The main purpose of the preprocessing step is to align the images, and crop them in two images representing both hemispheres. To this end, the preprocessing step consists of (see also Figure 3): (1) Diffeomorphic registration of the input subject image to a CTA atlas (Peter et al., 2017), in which the MCA region is known for both hemispheres. A multi-step affine plus diffeomorphic registration was applied using the ANTs toolkit (Klein et al., 2009). This registration implicitly includes the resampling of the images to a 1 × 1 × 1 *mm*^3^ resolution. (2) Flipping of the input image, depending on the affected hemisphere, in order to have the affected hemisphere always at the right side of the input image. (3) Separation of the image in two symmetrical sub-images containing the hemispheres. Here a small overlap of 10 voxels was used to make sure each sub-network has enough context to work with. (4) Mirroring the left (healthy) hemisphere to obtain voxel-wise correspondence with the affected hemisphere; (5) normalizing the intensity values in a range [−1, 1]. The normalization was performed to have the image values of between 35 and +165 Hounsfield units falling in the given range and clipping values outside the range to −1 (if below 35) and +1 (if above +165). The normalization interval was initially chosen by visual inspection, to optimize the contrast between brain tissue and the visibility of the vessels.

#### 2.1.4 Siamese sub-network

**Figure 4:**
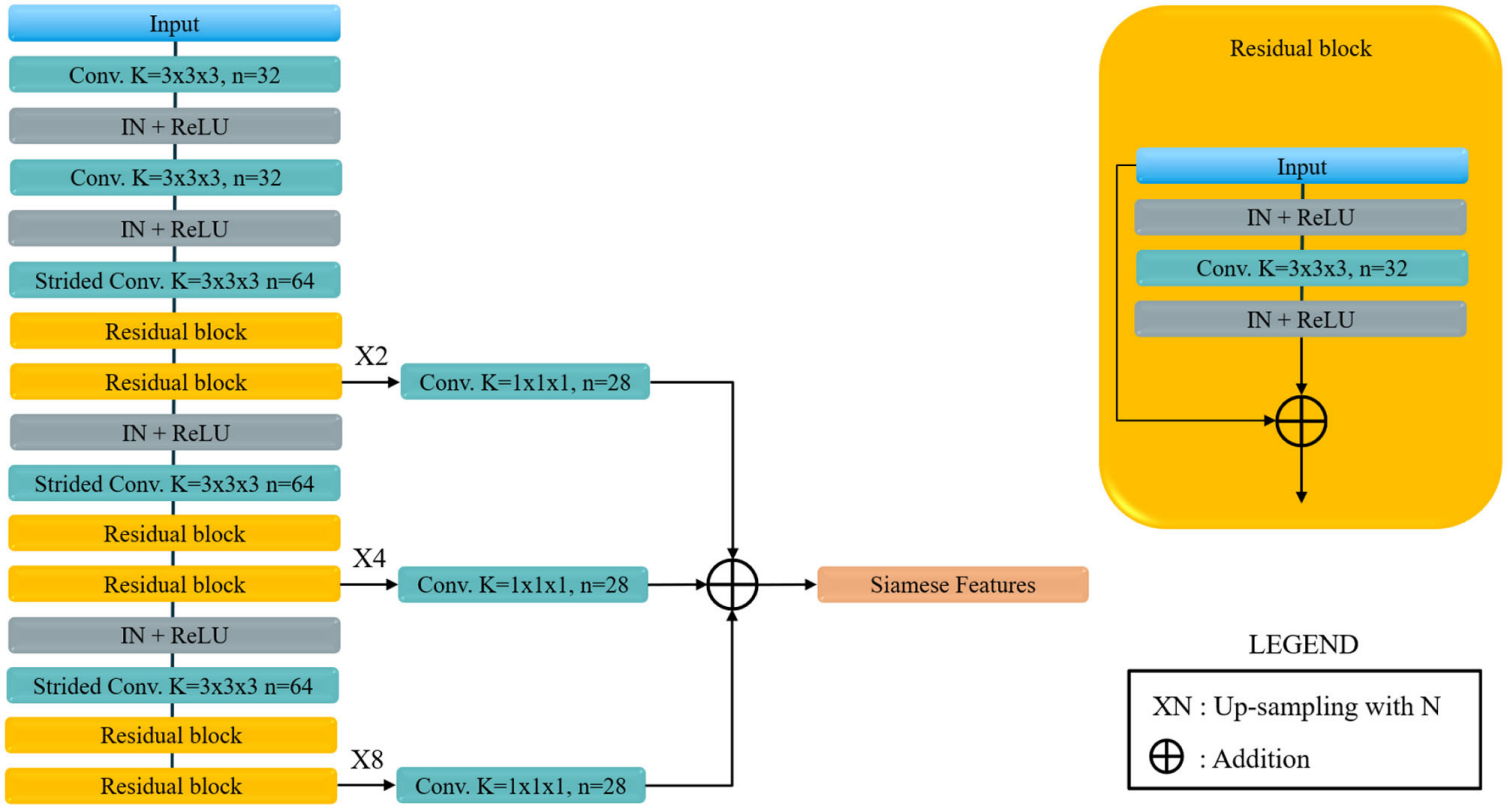
Voxel Residual Network used as Siamese sub-network. Conv. stands for convolution, Strided Conv. indicates a covolution with stride 2 in all directions, K is the kernel size, *n* the number of features, IN stands for Instance Normalization and ReLU for the rectified Linear Unit non-linear activation. Siamese feature are the feature map for each of the Siamese sub-network, that are later joined to obtained the final classification.

For the sub-network of the SNN model we adapted the VoxResNet (Chen et al., 2018) model architecture. This architecture was selected because of two reasons. First it has excellent performance which are, from our experience, similar to the performance of the more popular and widely used 3D U-Net (Çiçek et al., 2016). And secondly it has the advantage of a less computationally demanding decoding component when compared to the aforementioned U-Net. The adapted VoxResNet architecture used in this paper is shown in Figure 4. Since for our application we do not need a high-resolution volumetric response from the network, the decoding components generate an output image down-sampled space (with a factor of two). The other relevant difference when comparing to the original architecture is that we used Instance-Normalization instead of Batch-Normalization which provides better performance in case of training using unit-sized mini-batches (Çiçek et al., 2016; Ulyanov et al., 2017). The volumetric feature map output of the VoxResNet is then masked with the MCA label image, available from the CTA atlas, and reduced to a hemisphere-wise feature activation vector using global average pooling. The MCA masking provides the network with an extra input on the region of interest to be used for Collateral Scoring.

#### 2.1.5 Siamese feature joining

The feature activation from the MCA regions of the two hemispheres are then concatenated and fed to a classification layer with a softmax activation that output the four-values Collateral Score. The proposed architecture permits generating a Class Activation Map (CAM) (Zhou et al., 2016), which enables interpretability of the output score of the network model, by inferring the model again with the same input but without including the global pooling layer. In this way the feature vector will be replaced by a volumetric output for each Siamese feature which will propagate to the output to obtain a voxel-wise activation for each class.

#### 2.1.6 Training procedure

The SNN was trained end-to-end using back-propagation and the available four-values collateral score as reference. Mini-batch learning was used, with a mini-batch size of 1 in order to train the model on a single GPU^3^ and using Adam as stochastic optimizer (Kingma and Ba, 2014). The loss used for training is a combination of binary and categorical-entropy where dichotomized and four-valued collateral scores are used as target values. Note that dichotomized prediction can be directly computed from the four-valued prediction for each sample by simply summing the predictions of the last two classes. The final loss is given by a weighted combination of these losses:


$$\begin{array}{l} ℒ\;=\;(1-\alpha )[-(y_{2,3}\log(p_{2,3}))-(1-y_{2,3}\log(1-p_{2,3}))]-\\ \;\alpha \sum_{c=0}^{3}(y_{c}\log(1-p_{c}))), \end{array}$$

where *p*_*c*_ indicates the model prediction for score class c and *y*_*c*_ the reference score class. With *p*_2, 3_ and *y*_2, 3_ we indicate respectively the dichotomized prediction (prediction for combined score classes 2 and 3) and the dichotomized reference score. This loss allows to re-define the problem as a classification even if the classes are ordinal. That is because the dichotomized loss penalizes the mislabeling of more than 1 ordinal class (0 to 2, 3, or 1–3 or vice-versa).

For the optimization we used a warm-up learning rate scheduler (Smith, 2017) using a single cycle which allows us to stabilize the training over different training subsets which were used in our experiments (see Section 3). Specifically we defined a scheduler to linearly increase the learning rate for half of the training cycle and then decrease that until zero with a cosine-like decay. Since the manual collateral score is relatively noisy we used label smoothing, with a smoothing *s* of 0.2, to decrease the negative impact of label uncertainty and increase classification performance (Müller et al., 2019). A smoothing of 0.2 has the approximate effect of decreasing the k-hot encoded reference label values of 0.2 (from 1.0 to 0.8) for each output class, and therefore decrease the confidence of said reference labels:


$$\begin{array}{l} \begin{array}{c} y_{s}=\left(1-s\right)*y+s/K \end{array} \end{array}$$

where *y* is the k-hot encoded reference label, *y* its smoothed version and *K* is the number of classes (5 in our experiments). Since we have a relatively small set of training data affected by a large class imbalance we used substantial augmentation combined with sample balancing during training. The number of samples for each class was set to be the same at each epoch by up-sampling the less represented classes and down-sampling the more represented. Then each sample was augmented randomly using a random rotation, translation, scaling and elastic (B-Spline based) augmentation.

#### 2.1.7 Post-processing

During the evaluation of the collateral score results a post-processing step is performed to adapt the classifier threshold so that the operating point of the classifier is similar to the one of the human observer. We used the sensitivity of the human observers (taken from the inter-observer variability of the score) in scoring the dichotomized collateral to obtain the desired threshold θ to be applied to the trained classifier. This threshold was used both to obtain the predicted dichotomized score and to obtain the four-values score: the dichotomized score at the provided threshold was used to find the subset of classes in the four-values score (e.g., 0, 1 or 2, 3); then the subclass within this subset was selected according to the multi-class prediction. This post-processing correction was used because the dichotomized labels are more reliable than the four-values scores (see Section 2.2.1).

### 2.2 Data

Two different sets of data were used in this study. CTA images from the MR CLEAN Registry were used as a training and validation set, and the CTA images from the MR CLEAN Trial (Berkhemer et al., 2016) were used as an independent test set. The MR CLEAN Registry is a registry of stroke patients who underwent endovascular therapy from March 2014 till December 2018. This registry contains images from a large variety of hospitals in the Netherlands, and from a large variety of vendors. From the MR CLEAN Registry, 347 subjects were randomly selected for development, training and validation of the method, after applying selection criteria on slice spacing and thickness (< 1.5 mm), contrast phase (peak arterial, equilibrium, and early venous), image quality (good image quality according to core lab) and brain coverage (>50%), similar to Su et al. (2020). Initially the development set was split in a training set (278) and a validation set (69) which was kept as a last resource to validate the algorithm performance before the final evaluation on the MR CLEAN Trial set. The split was done by stratifying the subject cases according to the collateral score and the vendor of the CT scanner used for image acquisition. For the independent testing, the MR CLEAN Trial dataset was used. From the 495 subjects with available CTA images, we selected all cases with at least a 50% brain coverage (425).

#### 2.2.1 Inter-observer variability

Two subset of the MR CLEAN Registry and the MR CLEAN Trial datasets were selected to evaluate the inter-observer variability of the collateral score: (1) a sub-set of MR CLEAN registry data, and a sub-set of the MR CLEAN data with the characteristics showed in Table 1. The procedure to obtain the MR CLEAN Registry inter-observer set is described in Su et al. (2020): for 269 subject of the MR CLEAN Registry dataset two independent raters scored the CTA images to evaluate the score variability resulting in an inter-observer multi-class accuracy of 0.64 for the four-value scores, an accuracy of 0.81, a sensitivity of 0.81, and a specificity of 0.85 for the dichotomized score.

**Table 1:** Dataset description.

		**MR CLEAN Registry**			**MR CLEAN**	
**Properties**	**Category**	**Training**	**Validation**	**Entire**	**Test**	**Inter-observer**
Vendor	GE	17	2	19	130	16
	Philips	81	21	102	71	3
	Siemens	165	41	206	162	7
	Toshiba	15	5	20	62	13
Collateral	0	12	4	16	21	5
	1	82	21	103	145	13
	2	123	29	152	122	10
	3	61	15	76	137	11
Slice thickness [mm]	[0.5–0.75)	244	59	303	97	26
	[0.75–1.00)	10	3	13	94	13
	[1.00–1.5)	24	7	31	146	0
	[1.5–2.0]	–	–	–	88	0
Voxel spacing [mm]	Range	0.34–0.84	0.36–0.90	0.34–0.90	0.23–0.79	0.38–0.70
	Average	0.48	0.48	0.47	0.48	0.53
Gender	Females	131	33	164	177	14
	Males	147	36	183	248	25
Subjects	Total	278	69	347	425	39

For the MR CLEAN Trial inter-observer set all the images were rated on three characteristics used for the selection: CS (0/1/2/3), image quality (good/intermediate/bad) and location of occlusion [Internal Carotid Artery (ICA), Middle Cerebral Artery, horizontal segment (M1) and insular segment (M2)]. The selection was aimed to include 39 scans which are qualitatively suitable for radiology training in collateral scoring. Because the MR CLEAN Trial is a multicenter trial, image quality, and characteristics differ between cases and a quality selection is needed to make collateral scoring as accurate as possible. For this set all the images were rated by multiple observers with different level of experience: medical doctors (MD), radiologists and medical student at different year of study. The inter-observer accuracy of the four-values score varies from a minimum accuracy of 0.62 to maximum accuracy of 0.67 depending on the level of the observers with an average accuracy between all observation of 0.65. For the dichotomized score the minimum and maximum accuracy per observer sub-set are 0.87 and 0.89 with an average accuracy of 0.89.

## 3 Experiments and results

### 3.1 Experiments overview

For optimizing the network and hyperparameters of the deep learning model a cross-validation scheme was adopted using the training set. The training set was split in five-folds using the same stratification criteria that were used to split the MR CLEAN Registry into the training and validation set (see Section 2.2). The best performing method architecture and hyper-parameters were selected by evaluating the performance on the validation folds. Considering the architecture design and hyperparameters, we chose to keep the Voxel Residual model as in the original paper and we set the Siamese features as many as the GPU memory allowed, that was 28 features. To define the best training strategy we started with a training a model to predict dichotomized collateral score and then extended to the four-values score as explained in Section 3.2. Afterwards the training was stabilized across the different folds to obtain an optimal number of epochs to train the final model. Then an ablation study was performed to verify that the proposed design choices are optimal. Next, we also investigated ensembling of model predictions to further improve the classification performance. Based on these results, a final model was trained on the complete training set from the MR CLEAN Registry, and assessed on the MR CLEAN Trial images, both for collateral scoring, and for using the collateral scores as input to a state-of-the art prediction model for treatment outcome prediction in acute ischemic stroke. Each of these steps is detailed in the following sections.

### 3.2 Dichotomized and four-valued collateral score prediction model

**Figure 5:**
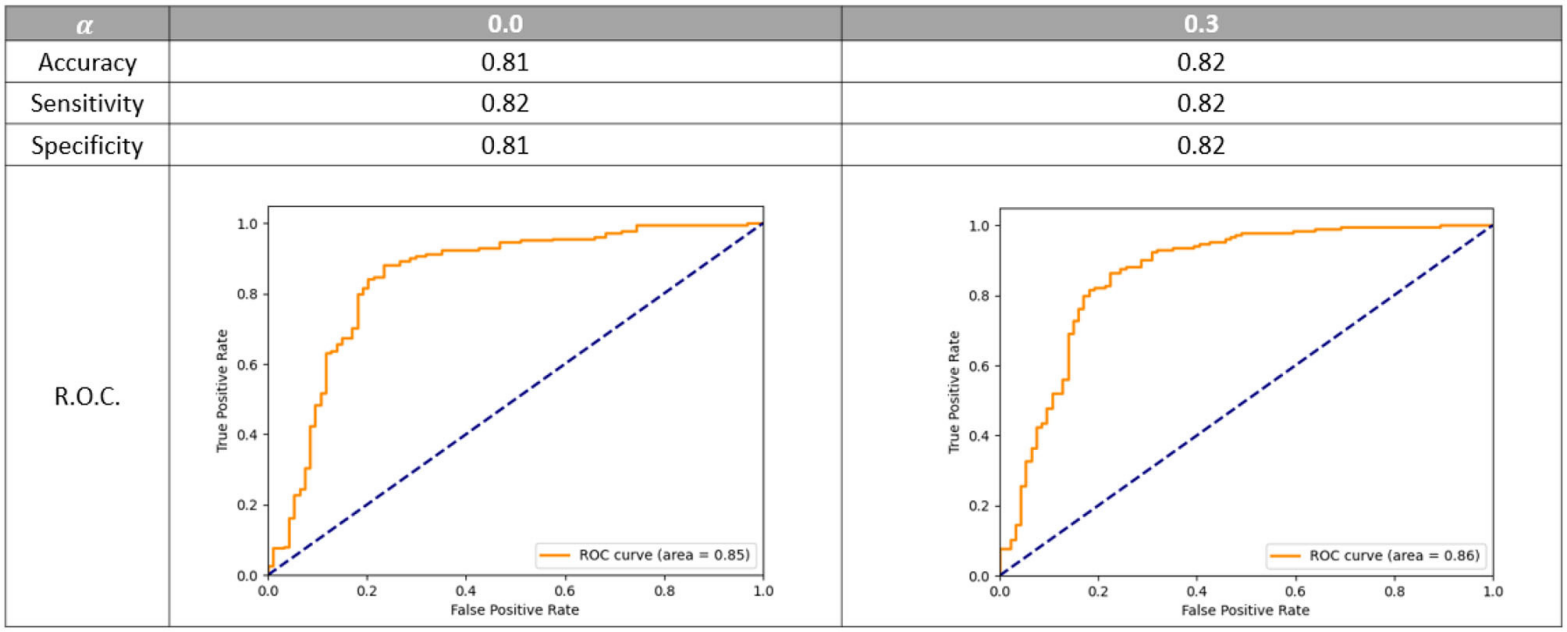
Dichotomized accuracy, sensitivity, specificity, and R.O.C. using different value of α. R.O.C. is the receiver operating characteristic curve. The models are evaluated on the epoch that gives on average the best multi-class accuracy on the validation set.

We first designed the network architecture and fine-tuned optimal hyper-parameters for the prediction of a dichotomized collateral score. This was done by setting the α parameter to 0 in the hybrid loss (1). Next we performed experiments to tune the parameter α and we introduced label smoothing to alleviate the issue of having noisy labels. In Figure 5, the dichotomized score performance is shown for the α equal to zero and the optimal α of 0.3. For this value of α the model trained to predict the four-valued collateral score can perform slightly better than a model trained to predict the dichotomized score.

### 3.3 Training stabilization

**Figure 6:**
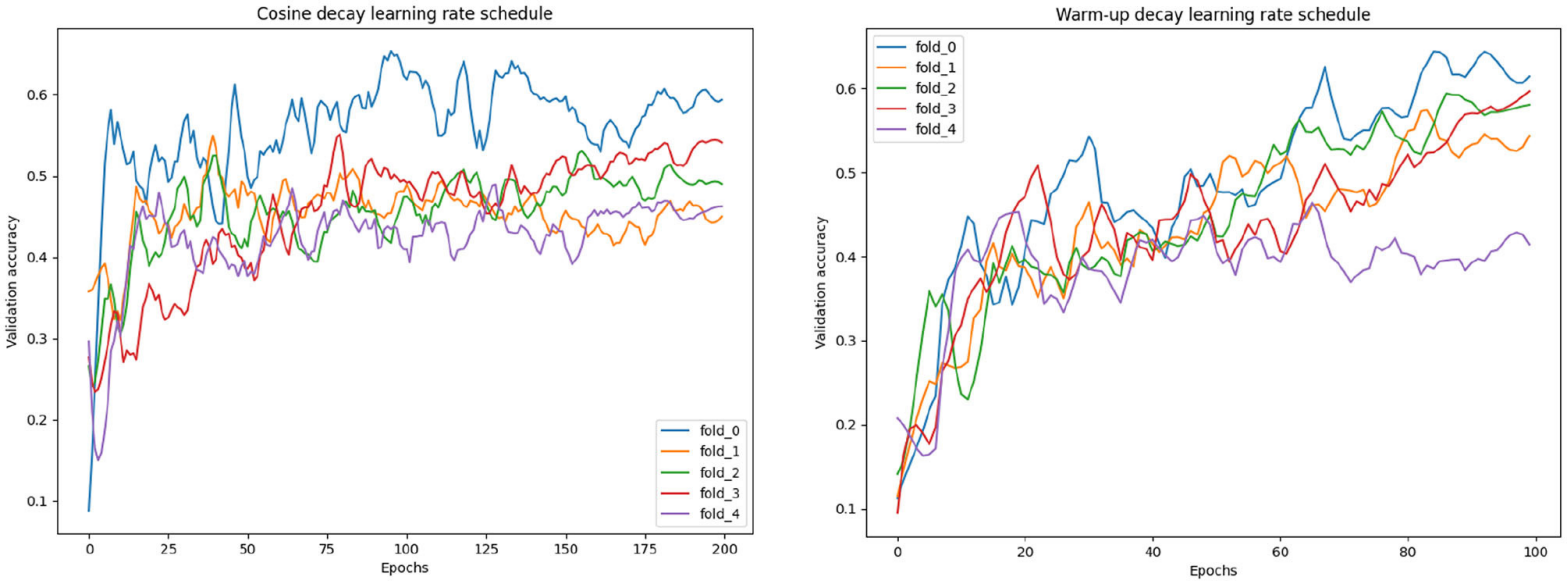
Validation curves with different learning rate schedules.

When running the cross-validation experiment we found that the training of the network was relatively unstable in the sense that the model was converging to the best validation loss at quite different epochs for each fold. Since, as explained later in this Section, we want to obtain an optimal number of epochs for training of the final model, we need the training to converge uniformly among different folds. To stabilize the model convergence across different training subset, i.e., the cross-validation folds, a warm-up learning rate schedule was used. In Figure 6, we compared the validation accuracy variation during training when using the warm-up learning rate schedule with the validation accuracy when using a regular learning rate decay. For the warm-up scheduler the following parameters were set: α = 0.3, maximum learning rate at half training 0.0002 and 100 epochs. For the learning rate decay scheduler the following parameters were set: α = 0.5, starting learning rate 0.0005, 200 epochs and a cosine shaped decay schedule. Considering the performance of the models corresponding to the best validation metrics, the models perform similarly with a score accuracy of 0.62. However, considering the validation accuracy when selecting the training epoch that gives the highest average accuracy across all the folds we have that using warm-up decay gave an improved accuracy of 0.56 while the cosine decay model an accuracy of 0.54. Therefore, the warm-up decay lead to a more stable training across the different folds.

### 3.4 Hyperparameters

After our experiments, the hyperparameters were set for the candidate models (as shown in Figure 4) to be used for the ensemble. Twenty-eight Siamese features, used to represent each hemisphere, were used; the multi-class weight loss α was set to 0.3; the label smoothing was set to 0.2; we used the Adam optimizer with a warm-up learning rate scheduler with maximum learning rate at half training of 0.0002, and starting (and final) learning rate of 0.0, and 100 epochs; for augmentation we used a random translation of 10 voxels in-plane and three voxels in the slice direction, a random rotation between −20° and 20° for all planes and, for the elastic augmentation, a B-spline grid of 20 × 20 × 20 voxels was used with a random perturbation (per grid-point) of 10 voxels.

### 3.5 Ablation study

The training setting and strategy was verified by ablation. Since we used an established architecture the ablation is focused on verifying whether (1) the Siamese architecture, (2) the hybrid binary and multi-class loss, and (3) the learning rate warm up strategy were effective on improving performance. Note that we already verified in Section 3.2 that the hybrid loss was effective on obtaining performance similar to the binary loss for the dichotomized collateral score. Here we also verified that the multiclass performance are not reduced by the hybrid loss. As ablation, we run the cross-validation training with the same parameters as described in Section 3.4 but unsetting each of the methodology to ablate. In Table 2, the ablation results are shown. For all the results we evaluated the validation set (in a cross-validation fashion) using the model at the epoch that gave on average the highest performance across the five-fold. In this way we could also take into account the training stability that is the main reason why we selected the warm-up learning rate scheduling. Note that for the No-Siamese experiment the model capacity is the same as for the Proposed (Siamese) model and only the input and forward pass were changed to implement this architecture. The inputs were pre-processed as explained in Section 2.1.3 but without splitting the images in two hemispheres. The results show that ablating each of the proposed solutions degrades the performance of the model both in terms of multiclass score accuracy and dichotomized score metrics.

**Table 2:** Cross-validation performance of models trained using different settings: Selected—model trained with the selected parameters; No-warm-up—model trained without warm-up learning rate scheduler but just with a cosine annealing rate decay; Multi-class—model trained with multi-class loss without contribution of the dichotomic loss; No-Siamese—model trained using the entire brain as input, and no Siamese architecture.

**Model**	**Multiclass metrics**	**Dichotomized metrics**			
	**Accuracy**	**Accuracy**	**Sensitivity**	**Specificity**	**AUC**
Proposed	0.56	0.82	0.82	0.82	0.86
No-warm-up	0.54	0.81	0.83	0.76	0.85
Multi-class	0.51	0.72	0.82	0.70	0.82
No-Siamese	0.50	0.77	0.82	0.69	0.82

The models are evaluated on the epoch that gives on average the best multi-class accuracy on the validation set.

### 3.6 Training and evaluating the model ensemble

The last part of the model optimization process was the selection of a model ensemble: using the optimal setting found in the previous experiments, three models were trained using different random processes initialization. The cross-validation performances of the ensembles and of the individual models were evaluated and compared to each other. These results are shown in Table 3. The ensemble of three models has better evaluation metrics than any individual model when considering both multiclass and dichotomized metrics. Therefore, we used the ensemble as our selected method.

**Table 3:** Cross-validation performance of models trained using different random initialization seeds and of their ensemble.

**Model**	**Multiclass metrics**	**Dichotomized metrics**			
	**Accuracy**	**Accuracy**	**Sensitivity**	**Specificity**	**AUC**
Model A	0.62	0.82	0.84	0.79	0.86
Model B	0.61	0.81	0.82	0.79	0.83
Model C	0.60	0.79	0.82	0.76	0.84
Ensemble	0.61	0.83	0.84	0.82	0.86

AUC stands for Area Under the Curve.

**Figure 7:**
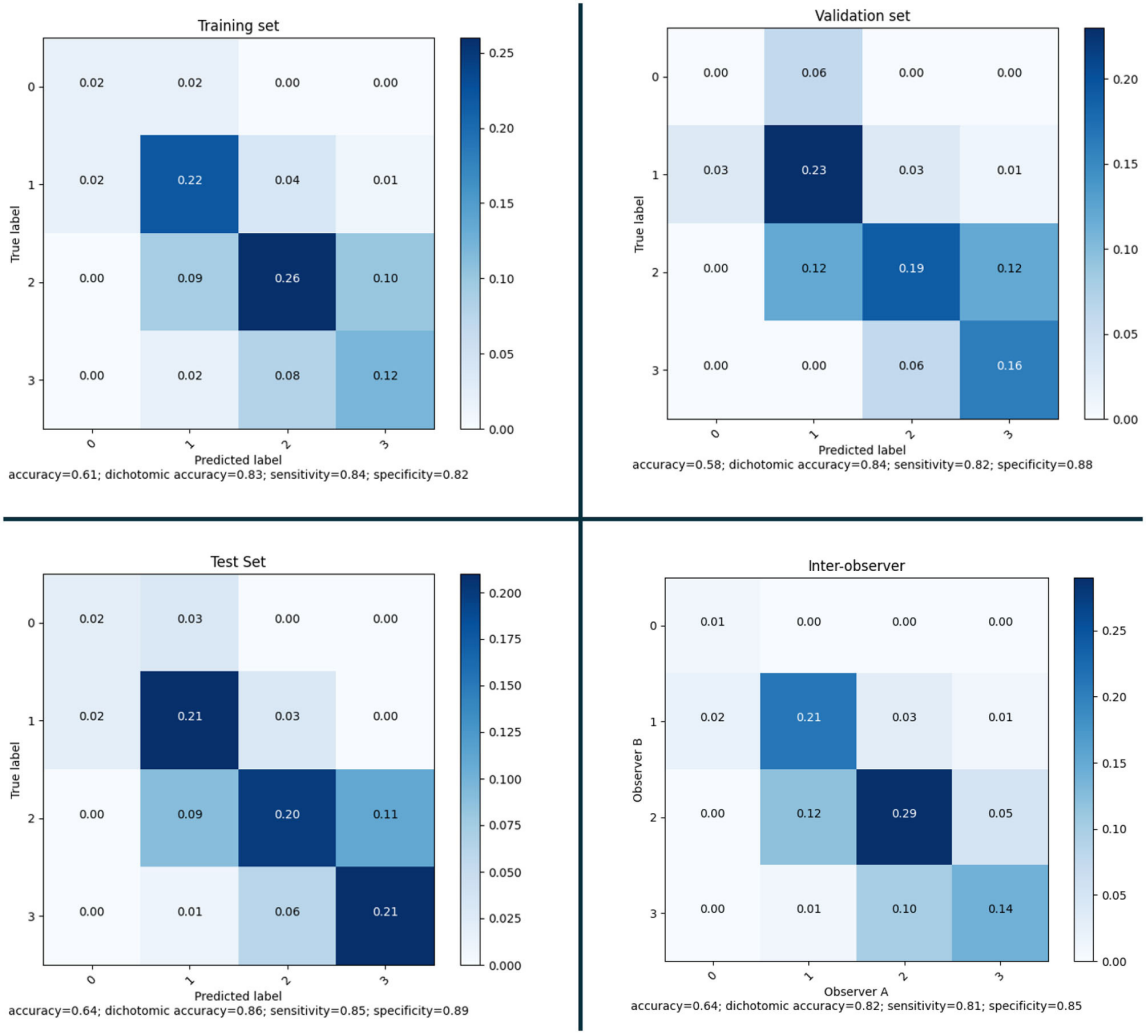
Confusion matrices and performance for the selected model on training set (according to the five-fold cross-validation), validation set and test set; and inter-observer variability.

**Figure 8:**
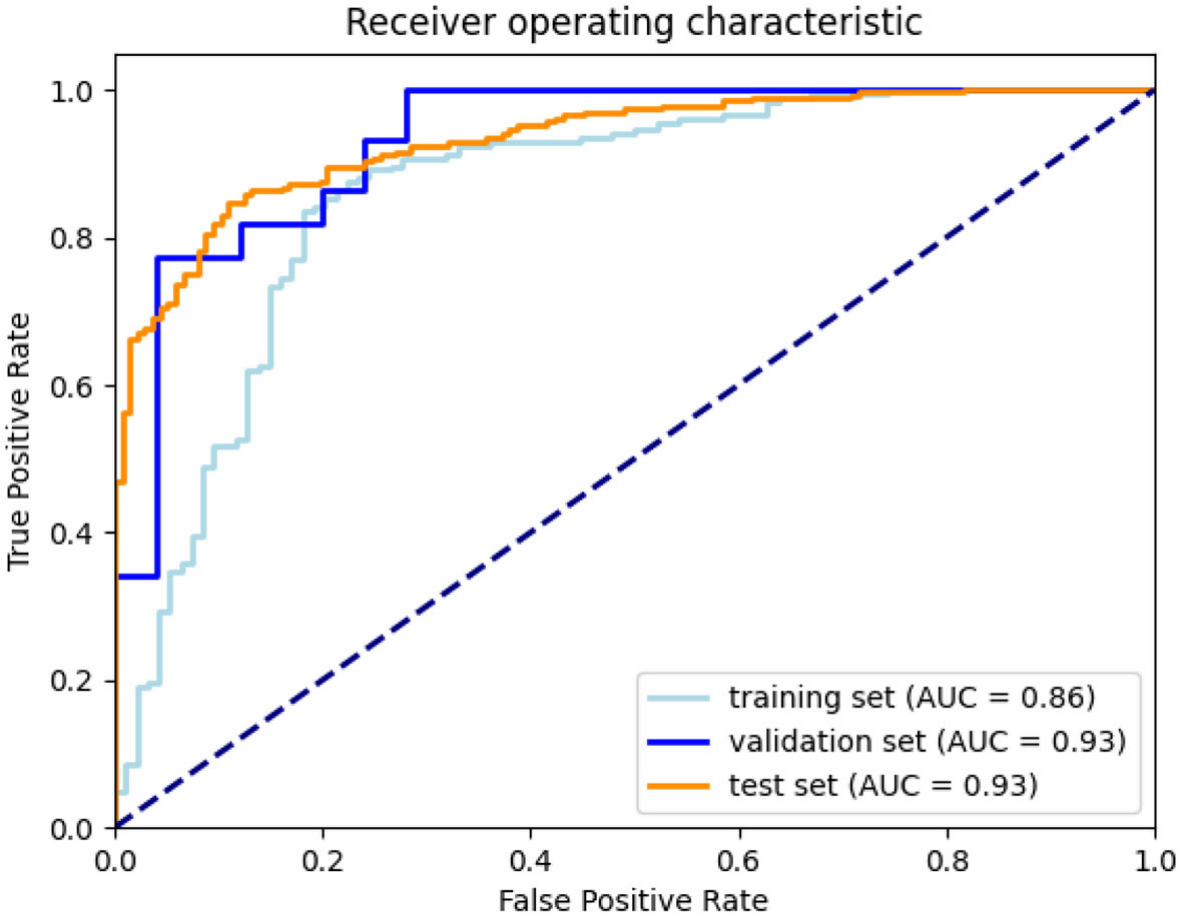
ROC curves for training (according to the five-fold cross-validation), validation, and test set.

After defining the final ensemble, we re-trained the model using the entire training set. In this training the number of epochs was selected as the epoch that gave the highest average validation multi-class accuracy over the five-fold's training. The model resulting from this final training was evaluated on the validation set and on the test set. Using the performance on the validation set and the inter-observer sensitivity the optimal threshold θ was computed as the threshold that resulted in a sensitivity equal to or higher than the inter-observer sensitivity. This optimal threshold of 0.53 was then used for the final evaluation on the test set. In Figure 7, the confusion matrices and performance metrics are given for the training, validation, test set and the inter-observer agreement. Multi-class accuracy, and dichotomized accuracy, sensitivity, and specificity are provided. The confusion matrices show that for all the datasets used for the evaluation the performance are close to the interobserver performance. The confusion with a score difference larger than one point is extremely rare, especially on the test set. The most confused scores are 2 and 3 and the least are 0 and 1. On the test set the confusion error is more balanced across the classes than the interobserver confusion and all the metrics show equal or higher performance than the interobserver. In Figure 8, the ROC curves are provided for the dichotomized collateral score for training, validation, and test set. Note that for the training set the metrics are related to the models with best validation accuracy for each of the cross-validation fold. The ROC curves of the dichotomized score classification of the validation and test set are similar and represent a high performing classifier.

**Figure 9:**
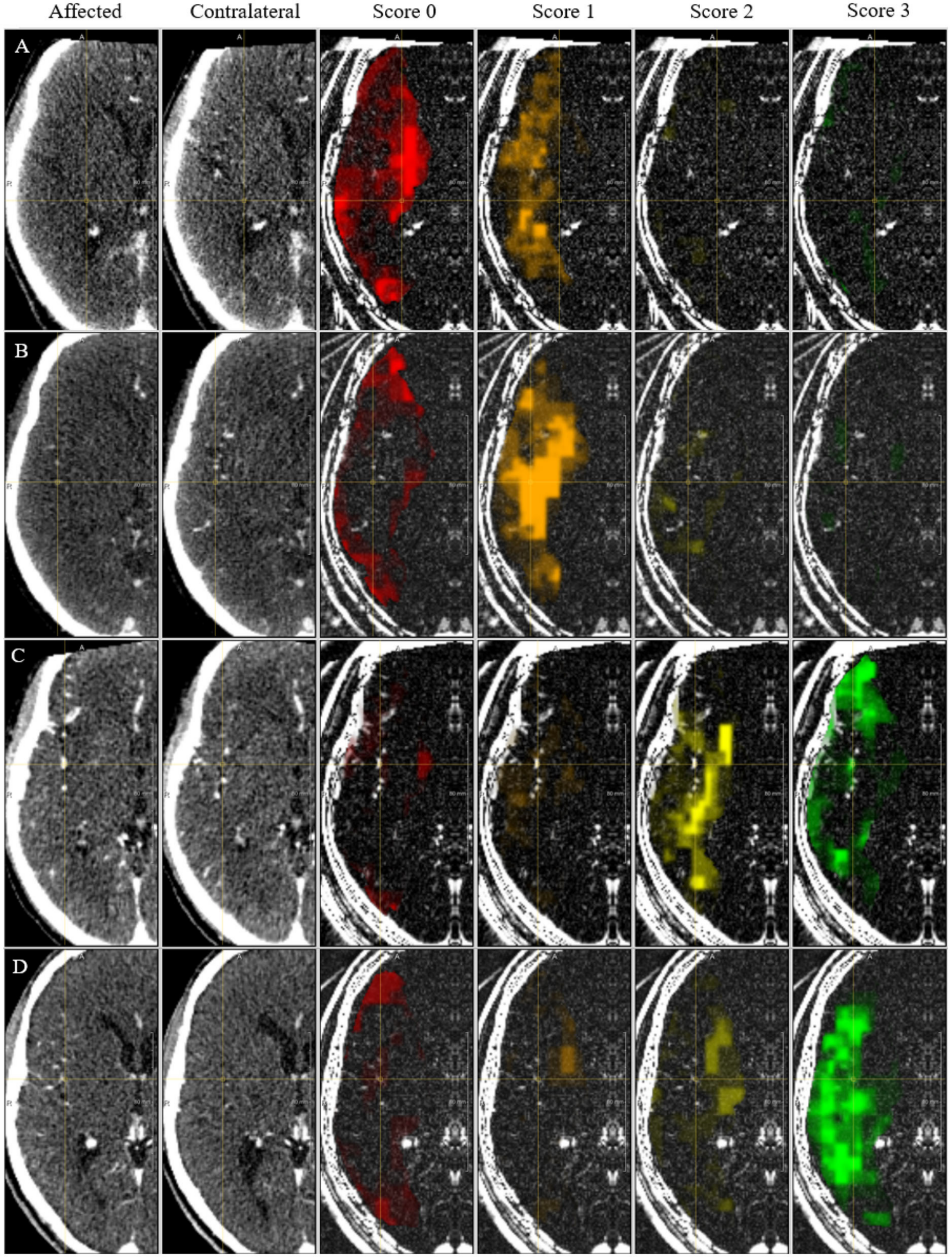
CAM for the collateral score. Each column shows the affected hemisphere, the contra-lateral side, the CAM for each score overlaid on the absolute intensity image. **(A–D)** Indicate cases with a correct prediction of collateral score of 0, 1, 2, and 3, respectively.

In Figure 9, we show CAMs for four example cases with correct predicted score. The CAM of the ensemble is the average of each model's activation map. The underlying images in figure have been pre-processed as the input images of the SNN model but without intensity normalization. For each of the scores a CAM is obtained and shown in the figure with a different overlay color. The maps are overlaid on the voxel-wise absolute difference image between the affected and healthy hemisphere so that is easier to visualize vessels that are only on one hemisphere. The CAMs show that the model focus on the image regions where the vessel signal is different between the two hemispheres: for the score 0 and 1 the regions in the affected hemisphere that have lower contrast enhancement with respect to the contralateral hemisphere are highlighted while for the score 2 and 3 the regions with higher contrast enhancement are highlighted.

### 3.7 Assessment on test set

**Figure 10:**
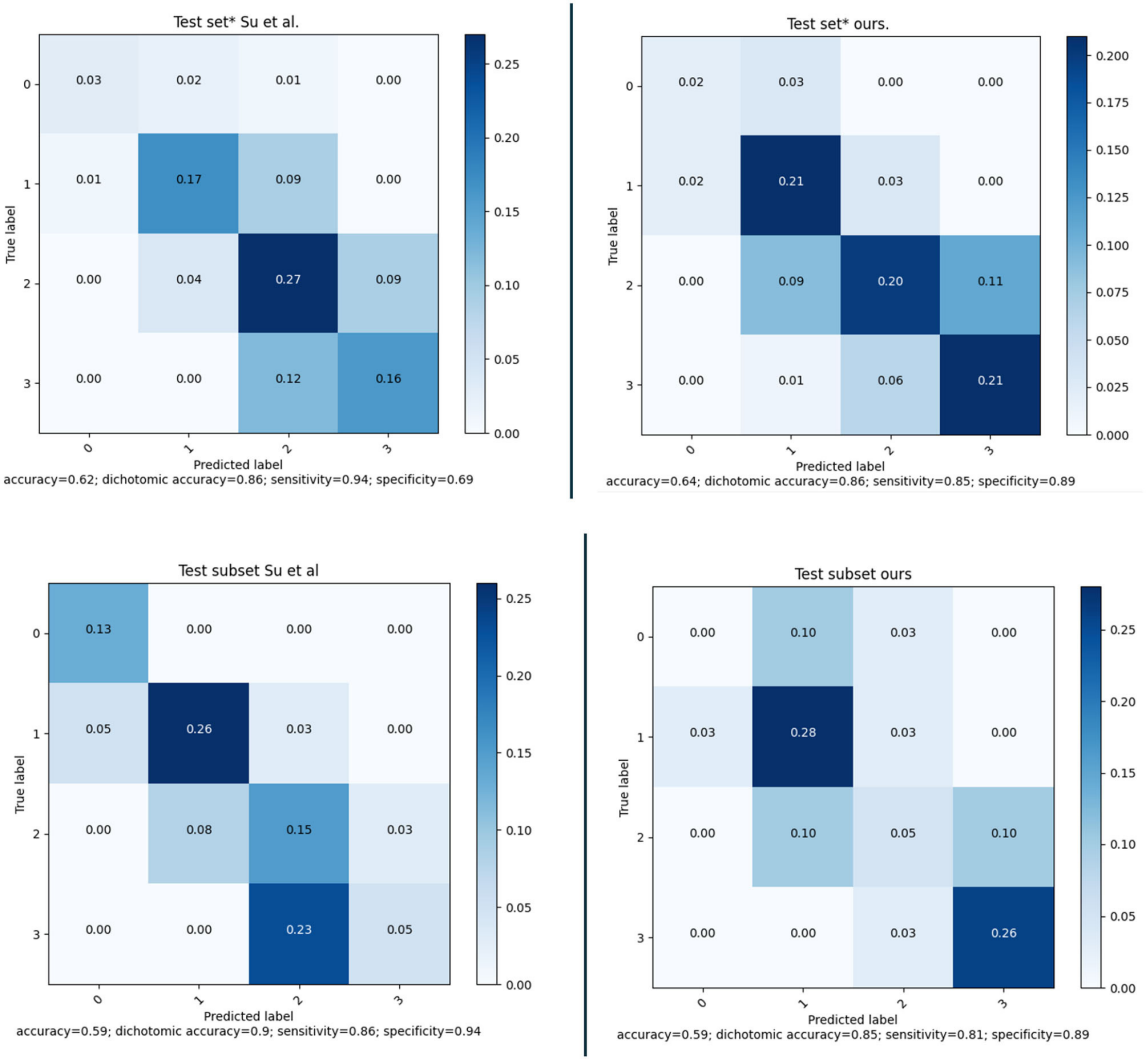
**(Top)** Confusion matrices and performance of our method and of the method of Su et al. evaluated on a subset of 418 subject of the MR CLEAN Trial dataset. **(Bottom)** Confusion matrices and performance of our method and of the method of Su et al. evaluated on a subset of 39 subject of the MR CLEAN Trial dataset.

The MR CLEAN Trial dataset was used as an independent test set for the method developed. The final model was trained as explained in Section 3.2. From the MR CLEAN Trial dataset, 425 subjects had a CTA with more than 50% brain coverage. A collateral score was computed for each of these subjects. These collateral scores were compared with the human annotated collateral scores, and with the method of Su et al. (2020). Since, Su et al. (2020) have different selection criteria for the input data the methods were compared for the common subset of the MR CLEAN Trial dataset that consists of 418 subjects. The results are in Figure 10 (top row). The results show that the proposed method have similar performance than Su et al. (2020) on the entire test set and slightly worse performance on the small test set.

**Figure 11:**
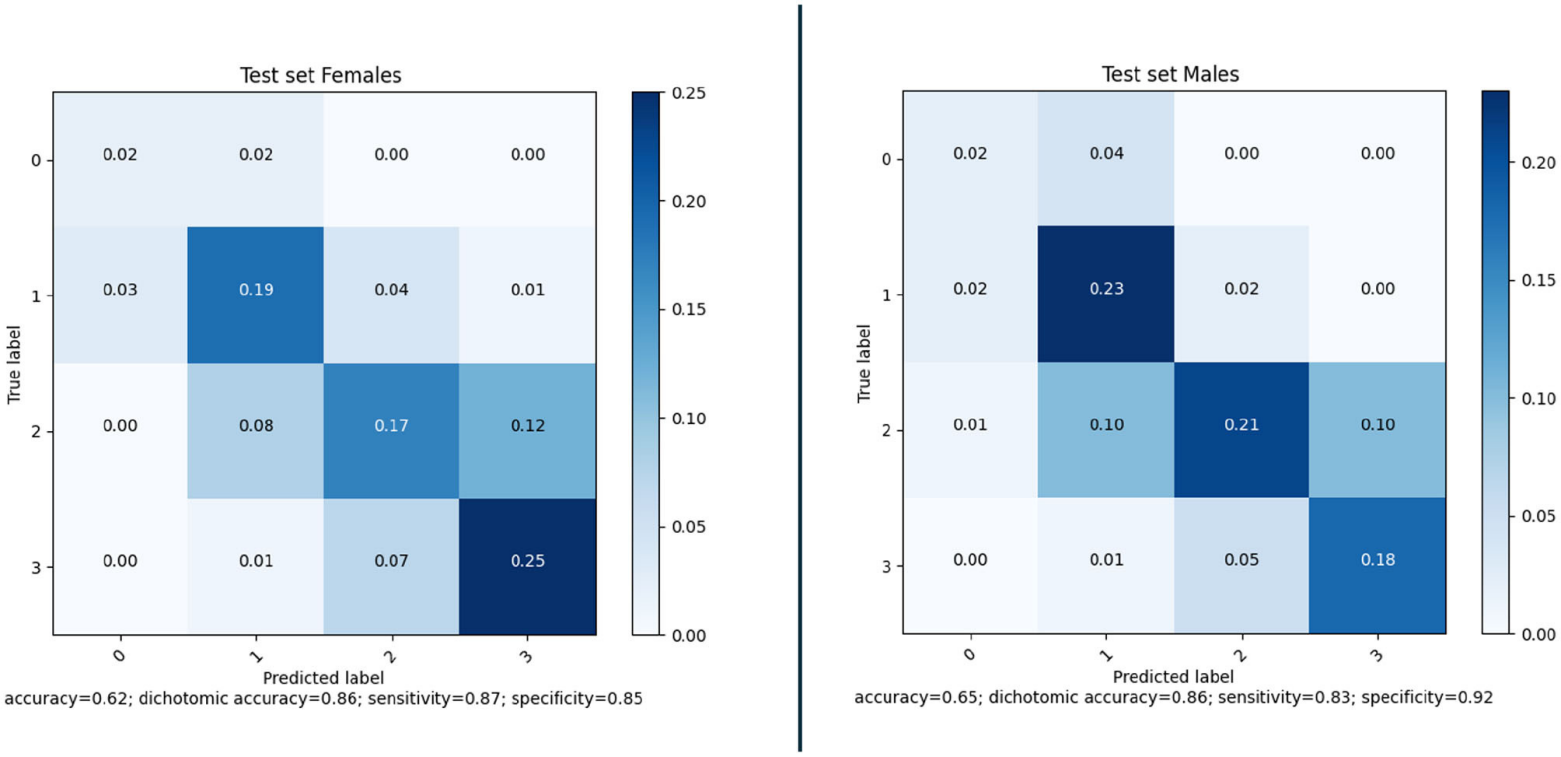
Confusion matrices and performance of our method for Female (177) and Male (248) subjects of the MR CLEAN Trial dataset.

Awareness of gender differences in medicine is growing, and in particular for cardiovascular diseases it is becoming important to discriminate between males and females. In line with this, we also investigated whether there were differences between the performance of the model when applied to males or females. The sex-based performance is given in Figure 11; the results show that there is no significant difference in the results for males and females.

The same model ensemble was also used to predict the collateral score for the MR CLEAN inter-observer subset of 39 subject. The performance metrics of the model's ensemble on this dataset are shown in Figure 10 (bottom row) in comparison with the method of Su et al. (2020).

### 3.8 Application to prediction of treatment outcome

In addition, the collateral scores have been used as replacement of human annotated collateral scores in the MR Predicts functional outcome prediction model (Venema et al., 2017). This is a model that predicts positive functional outcome (modified Ranking scale < 3), as well as the modified Ranking scale at 90 days, from 11 clinical parameters, among which radiological biomarkers such as the Alberta Stroke Program Early CT Score and collateral score. The main purpose is to assess to what extent these collateral scores can be used in such models, and whether quantitative measures improve the prediction. The area under the curve of four different models [one trained with the human scored CS, one with the quantitative collateral score computed by Su et al. (2020), one with the collateral score from the SSN model, and one with a quantitative collateral score from the SSN model, where the quantitative value is the expectation of the collateral score, i.e., the weighted average of the four probabilities for the collateral categories output by the final layer of the SNN] is shown in Table 4. Note that in order to compare with the performance of Su et al. (2020). the evaluation was performed on the subset of 418 subject for which both method could be used. The results show that using the score and quantitative value from the proposed method slightly increases the AUC of the treatment outcome prediction model.

**Table 4:** Area under the curve for prediction of positive functional outcome on the 418 subject for which both the SNN and the method by Su et al. (2020) could be used.

**MR predicts using CS from:**	**Prediction of 90-days outcome (95% CI), AUC**	
	**mRS** ≤ **2**	**mRS 0–6**
Human obs	0.795 (0.757–0.833)	0.749 (0.711–0.787)
Su et al.	0.798 (0.759–0.837)	0.752 (0.713–0.791)
SNN categorized	0.800 (0.754–0.847)	0.749 (0.702–0.795)
SNN quant.	0.814 (0.777–0.851)	0.760 (0.723–0.797)

## 4 Discussion

We have presented an end-to-end collateral scoring approach. The model uses a Siamese Network approach that facilitates comparing features extracted from the affected and healthy hemispheres. Experiments with large clinical datasets demonstrate that such approaches can replace human scoring, and also can compete with approaches that mimic the human assessment.

In our experiments we showed that using an hybrid loss, which combines the four-values cross-entropy loss with the dichotomized binary loss, was effective in obtaining optimal performance for the dichotomized even when applied to the four-values classification. This loss was necessary because the standard categorical cross-validation counts all the classification error as equal while in our task the errors between classes far from each other is more relevant and should therefore be more penalizing to the loss. We expect that the standard categorical cross-entropy can be directly used without loss of performance in case of a significant increase of the training set available (Muhamedrahimov et al., 2021; i.e., several thousand cases). We also showed that using a warm-up learning rate schedule we could stabilize the training in order to obtain models performance uniform across different folds. Even though in Figure 6 we compared training experiments with slightly different parameters, the effect of these differences on the stability of the training is negligible. Therefore, we can say that the approach is effective in stabilizing the training and allows setting an optimal training strategy to be used in the final training. Looking at validation loss over different training epochs in Figure 6 we can notice that the curve is quite noisy. This is not surprising considering that we used a mini-batch size of one sample to overcome memory constraints. This effect could be mitigated by using more mini-batches and changing the training procedure using (1) a GPU with more memory, (2) multiple GPUs, and gradient accumulation, or (3) mixed-precision training. At the moment we did not investigate these alternatives and we think it is not likely that this would lead to a significant improvement of the model performance.

The collateral scoring was assessed on two different sets: one small set (inter-observer set) composed of carefully chosen CTA images with good quality, and an even distribution of collateral scores, and a large set (test set) of representative clinical data. For the smaller set, the method performs slightly worse than the method of Su et al. This may be explained by the fact that the distribution of the collateral scores in this set is different from the distribution that is normally encountered in clinical practice. The number of subjects with a collateral score of 0 was 13%, whereas the percentage of collateral scores of zero in the training set was only 4.3%. From the confusion matrix in Figure 10 (bottom row) it is clear that the zero cases are one of the larger errors for the end-to-end trained model. Having a more explicit computation of collateral scores by explicit comparing the amount of vessels in both hemispheres may give a better prediction. Still, the accuracy for collateral scoring and dichotomized collateral scoring are in the same range as the inter-observer values.

For the larger test set, the results are slightly different. There, the accuracy for collateral scoring and dichotomized collateral scoring are similar, and also the confusion matrices are similar. Note that, for both automated methods, the performance is similar in both test sets. However, the inter-observer variation is much smaller for the smaller dataset. That may be caused by the selection criteria, where good image quality was a pre-requisite. The overall image quality in the smaller set likely is better than the image quality in the larger dataset, and apparently the lower quality of the images affects human scoring more than the scoring of algorithms that have been trained on representative datasets.

Collateral scoring plays a role in clinical decision making. Having good collaterals may prolong the time window in which intra-arterial treatment is still considered beneficial, as the collateral flow may prevent the brain tissue at risk from dying. It is therefore relevant to know whether automated collateral scoring can be applied in such contexts as well. To this end, we used the automated collateral scores in an existing outcome prediction model (Venema et al., 2017). This model was developed to determine the treatment benefit based on various clinical baseline parameters, including radiological biomarkers. One of the underlying hypotheses was that automated collateral scoring may remove inter-observer variability and therefore improve the performance of the prediction model. In addition, using a quantitative score may also improve the outcome prediction over a categorical score, as it allows the model to discriminate between various collateral scores in a more quantitative way. The results shown in Table 4 indeed suggest both trends: the area under the receiver operating curve (AUROC) increases when using automated scores vs human scores, and also increases when the categorical score from the SNN model is replaced with a quantitative value. Still, the increases are small, and not statistically significant, so more experiments are needed to assess whether these hypothesis hold. It is, however, clear from these results that human scores can safely be replaced with well-trained automated methods.

For the final assessment of the models we used an external dataset. In addition, both the training and validation sets are multi-vendor and multi-center, and are data that have been acquired in normal clinical care. Eighty-six percent of the CTA images from the large external validation set could be processed. The main reason for not being able to process the images was the lack of a usable CTA (cases with at least a 50% brain coverage). We expect that with newer data, and improved imaging protocols for stroke patients in acute setting, even higher percentages of usable CTA images can be obtained. Further assessment may still be needed for images from populations with different ethnicity.

Direct prediction of biomarkers such as collateral scores is more difficult than task that are commonly done in medical imaging, such as the segmentation of anatomical structures. In this manuscript, we proposed a Siamese network model with a VoxResNet backbone, to compute features from both hemispheres to predict a collateral score. The experiments demonstrate that collateral scoring in such a way is in the inter-observer range, and performs similar to more traditional approaches. In the future, we will build on these results, and investigate to what extent similar approaches can be used for, e.g., functional outcome prediction, a task where the relation between image and predicted value is even less clear.

## 5 Conclusion

Concluding, we presented an Siamese network model that was trained in an end-to-end fashion to predict collateral scores from CTA images of acute stroke patients. For dichotomized collateral scoring, and AUC of 0.93 is obtained on an external test set. Dichotomized accuracy is 86%, and accuracy for categorized score is 0.64. These numbers are in line with inter-observer results obtained on large datasets. Application of these scores in a functional outcome prediction model also results in an AUC of 0.790, which is not worse than the AUC obtained with collateral scores obtained by humans, 0.785.

## Data availability statement

The data analyzed in this study is subject to the following licenses/restrictions: The MR Clean Registry and MR Clean Trial data are not publicly available. Requests to access these datasets should be directed to www.mrclean-trial.org, www.contrast-consortium.nl.

## Ethics statement

Ethical approval was not required for the study involving humans in accordance with the local legislation and institutional requirements. Written informed consent to participate in this study was not required from the participants or the participants' legal guardians/next of kin in accordance with the national legislation and the institutional requirements.

## Author contributions

VF, LW, TW, and AL contributed to conception and design of the study. P-JD, JH, JM, RB, and WZ were involved in the data acquisition. VF performed the implementation and training of the network and wrote the first draft of the manuscript. VF and LW performed statistical analysis. All authors contributed to the manuscript revision, read, and approved the submitted version.

## Footnotes

^1^ http://dx.doi.org/10.2471/BLT.16.181636

^2^Code is available at https://gitlab.com/radiology/igit/accurate/siamese_model.

^3^NVIDIA GeForce RTX 2080Ti.
